# Genome Sequences of 104 Escherichia coli O157:H7 Isolates from Pigs, Cattle, and Pork Production Environments in Alberta, Canada

**DOI:** 10.1128/MRA.01320-20

**Published:** 2021-01-28

**Authors:** Peipei Zhang, Saida Essendoubi, Julia Keenliside, Tim Reuter, Kim Stanford, Robin King, Patricia Lu, Xianqin Yang

**Affiliations:** a Agriculture and Agri-Food Canada, Lacombe, Alberta, Canada; b Alberta Agriculture and Forestry, Edmonton, Alberta, Canada; c Alberta Agriculture and Forestry, Lethbridge, Alberta, Canada; d University of Lethbridge, Lethbridge, Alberta, Canada; University of Maryland School of Medicine

## Abstract

Genome sequences of Escherichia coli O157:H7 originating from pigs are limited in the public databases. We sequenced 104 E. coli O157:H7 isolates from pig and cattle feces and pork production environments in Alberta, Canada. The information will aid studies investigating sources of E. coli O157:H7 contaminating pork and the associated environments.

## ANNOUNCEMENT

Shiga toxin-producing Escherichia coli O157:H7 can cause human disease ranging from self-limiting diarrhea to severe hemolytic uremic syndrome (HUS) (1–4). Cattle are a major reservoir of E. coli O157:H7 (5), and consequently, E. coli O157:H7 outbreaks attributed to contaminated beef are frequently reported (6). In contrast, published accounts have shown that the prevalence of E. coli O157:H7 in pigs is very low (7–19). However, three E. coli O157:H7 outbreaks (2014, 2016, and 2018) associated with pork in Alberta, Canada, have been reported (20–22). It was unclear whether the E. coli O157 isolates in the pork products were pig-adapted strains or due to cross-contamination from cattle. A surveillance study (23) and outbreak-related investigations recovered Shiga toxin gene-containing O157:H7 from pig fecal samples and pork production environments in Alberta. This study sequenced the genomes of these isolates and O157:H7 isolated from cattle in Alberta to identify the potential source of O157:H7 contaminating pork. These isolates included E. coli O157:H7 recovered from pigs (*n* = 41; Pig01 to Pig41), pork production-related environments (*n* = 25; Env01 to Env25), and cattle (*n* = 38; Cat01 to Cat12, Cat14 to Cat19, Cat21 to Cat23, and Cat25 to Cat41) in Alberta, Canada (23–29) (Table 1). Pig isolates were recovered from either the cecal contents of pigs in pork processing facilities or pig feces from barns (23). Cattle isolates were recovered from cattle rectal grabs and feces from the floors of feedlot pens or transportation trucks (24–29). The environmental isolates included those that had been recovered from water (Env22), manure (Env21), a mouse fecal sample (Env16), and pig feces (Env17 to Env20 and Env23 to Env25) on farms; pig carcasses (Env06 to Env14) in pork processing plants; sponge samples from processing environments (Env03 and Env15); and pork (Env01, Env02, Env04, and Env05) from retailers (23). The presence of Shiga toxin genes in these isolates was confirmed using PCR methods as described in previous studies (23–29).

**TABLE 1:** Genome characteristics of 104 Escherichia coli O157:H7 isolates recovered from pigs, cattle, and pork production environments in Alberta, Canada

Isolate	Reference or source for isolates^*a*^	Isolation source	Total no. of raw reads	Approximate coverage by trimmed reads (×)	Assembled genome size (bp)	G+C content (%)	No. of contigs	*N*_50_ (bp)	No. of coding genes	GenBank accession no.	
										SRA	Genome
Cat01	24	Cattle feces	6,161,335	204	5,406,716	50.19	203	15,1274	5,126	SRR12593972	JACXCH000000000.1
Cat02	24	Cattle feces	6,181,796	204	5,372,624	50.3	194	146,648	5,100	SRR12593971	JACXCI000000000.1
Cat03	24	Cattle feces	6,431,179	214	5,370,569	50.3	201	158,564	5,099	SRR12593956	JACXCJ000000000.1
Cat04	24	Cattle feces	5,253,742	165	5,394,228	50.3	218	158,564	5,126	SRR12593881	JACXCK000000000.1
Cat05	24	Cattle feces	5,681,813	186	5,283,362	50.3	172	157,968	4,980	SRR12593870	JACXCL000000000.1
Cat06	24	Cattle feces	5,695,423	191	5,302,207	50.3	161	149,125	5,003	SRR12593943	JACXCM000000000.1
Cat07	24	Cattle feces	5,246,946	174	5,341,015	50.29	173	157,969	5,055	SRR12593912	JACXCN000000000.1
Cat08	24	Cattle feces	5,787,961	192	5,406,390	50.24	187	157,855	5,142	SRR12593901	JACXCO000000000.1
Cat09	24	Cattle feces	6,369,653	209	5,432,093	50.34	181	188,185	5,136	SRR12593930	JACXCP000000000.1
Cat10	24	Cattle feces	5,610,289	174	5,433,395	50.34	174	188,185	5,140	SRR12593919	JACXCQ000000000.1
Cat11	24	Cattle feces	6,294,163	205	5,339,787	50.3	181	149,125	5,044	SRR12593970	JACXCR000000000.1
Cat12	24	Cattle feces	5,840,896	184	5,320,499	50.29	167	188,185	5,027	SRR12593965	JACXCS000000000.1
Cat14	24	Cattle feces	5,550,948	186	5,341,635	50.27	183	148,373	5,058	SRR12593964	JACXCT000000000.1
Cat15	24	Cattle feces	5,498,559	183	5,379,913	50.31	178	149,902	5,110	SRR12593963	JACXCU000000000.1
Cat16	24	Cattle feces	6,947,687	232	5,469,389	50.31	173	159,924	5,207	SRR12593962	JACXCV000000000.1
Cat17	24	Cattle feces	5,651,228	187	5,333,473	50.28	176	149,902	5,041	SRR12593961	JACXCW000000000.1
Cat18	24	Cattle feces	6,042,478	203	5,336,104	50.29	180	149,902	5,044	SRR12593960	JACXCX000000000.1
Cat19	24	Cattle feces	5,752,161	191	5,412,991	50.3	221	147,698	5,150	SRR12593959	JACXCY000000000.1
Cat21	24	Cattle feces	6,624,682	216	5,340,590	50.29	171	151,080	5,058	SRR12593958	JACXCZ000000000.1
Cat22	25	Cattle feces	5,416,513	170	5,409,663	50.31	218	147,636	5,146	SRR12593957	JACXDA000000000.1
Cat23	25	Cattle feces	5,420,488	177	5,336,464	50.28	176	157,969	5,051	SRR12593955	JACXDB000000000.1
Cat25	25	Cattle feces	6,812,867	225	5,347,895	50.27	189	148,495	5,068	SRR12593954	JACXDC000000000.1
Cat26	25	Cattle feces	8,803,343	282	5,421,915	50.27	167	15,0416	5,160	SRR12593953	JACXDD000000000.1
Cat27	28	Cattle feces	5,332,323	176	5,360,243	50.29	200	147,636	5,072	SRR12593888	JACXDE000000000.1
Cat28	28	Cattle feces	6,718,640	223	5,367,096	50.29	204	148,495	5,080	SRR12593887	JACXDF000000000.1
Cat29	AAF	Cattle feces	5,957,708	190	5,397,794	50.29	158	149,125	5,109	SRR12593886	JACXDG000000000.1
Cat30	AAF	Cattle feces	6,120,909	201	5,466,098	50.2	202	148,373	5,213	SRR12593885	JACXDH000000000.1
Cat31	26	Cattle feces	7,256,603	244	5,437,015	50.31	194	148,373	5,167	SRR12593884	JACXDI000000000.1
Cat32	26	Cattle feces	5,319,024	169	5,320,934	50.29	171	157,969	5,029	SRR12593883	JACXDJ000000000.1
Cat33	26	Cattle feces	6,238,940	204	5,316,169	50.29	167	188,185	5,022	SRR12593882	JACXDK000000000.1
Cat34	26	Cattle feces	5,522,086	168	5,378,008	50.31	176	149,902	5,104	SRR12593880	JACXDL000000000.1
Cat35	27	Cattle feces	5,466,632	173	5,342,583	50.29	179	151,007	5,052	SRR12593879	JACXDM000000000.1
Cat36	27	Cattle feces	5,911,358	193	5,383,505	50.31	172	157,954	5,115	SRR12593878	JACXDN000000000.1
Cat37	29	Cattle feces	6,327,181	209	5,440,787	50.3	176	157,968	5,166	SRR12593877	JACXDO000000000.1
Cat38	29	Cattle feces	6,533,695	220	5,409,551	50.34	160	189,393	5,143	SRR12593876	JACXDP000000000.1
Cat39	29	Cattle feces	5,003,894	163	5,408,802	50.34	170	189,393	5,129	SRR12593875	JACXDQ000000000.1
Cat40	29	Cattle feces	6,521,926	218	5,335,567	50.29	185	149,902	5,043	SRR12593874	JACXDR000000000.1
Cat41	29	Cattle feces	5,306,685	168	5,442,177	50.31	176	157,967	5,169	SRR12593873	JACXDS000000000.1
Env01	AAF	Raw pork	6,218,151	206	5,328,566	50.29	179	157,974	5,033	SRR12593872	JACXDT000000000.1
Env02	AAF	Processed pork	6,669,985	212	5,312,222	50.3	165	210,214	5,003	SRR12593871	JACXDU000000000.1
Env03	AAF	Pork processing environment	5,048,014	169	5,312,342	50.3	167	210,214	5,005	SRR12593869	JACXDV000000000.1
Env04	AAF	Raw pork	6,158,441	202	5,309,028	50.29	163	210,214	5,011	SRR12593952	JACXDW000000000.1
Env05	AAF	Raw pork	6,737,233	225	5,313,732	50.3	165	210,214	5,008	SRR12593951	JACXDX000000000.1
Env06	23	Pig carcass	4,580,260	144	5,348,089	50.31	153	157,969	5,061	SRR12593950	JACXDY000000000.1
Env07	23	Pig carcass	6,721,212	217	5,419,603	50.36	185	159,739	5,160	SRR12593949	JACXDZ000000000.1
Env08	23	Pig carcass	5,728,672	195	5,372,825	50.27	197	157,855	5,096	SRR12593948	JACXEA000000000.1
Env09	23	Pig carcass	6,352,130	206	5,419,847	50.36	184	159,739	5,163	SRR12593947	JACXEB000000000.1
Env10	23	Pig carcass	4,915,715	166	5,419,859	50.36	192	157,969	5,157	SRR12593946	JACXEC000000000.1
Env11	23	Pig carcass	6,976,628	222	5,420,985	50.36	188	159,739	5,162	SRR12593945	JACXED000000000.1
Env12	23	Pig carcass	7,435,484	257	5,340,088	50.29	172	157,969	5,055	SRR12593944	JACXEE000000000.1
Env13	23	Pig carcass	6,364,677	204	5,340,978	50.29	171	15,7969	5,059	SRR12593942	JACXEF000000000.1
Env14	23	Pig carcass	6,258,189	192	5,338,618	50.29	178	149,902	5,050	SRR12593941	JACXEG000000000.1
Env15	AAF	Pork processing environment	5,723,730	182	5,385,993	50.33	173	159,924	5,113	SRR12593940	JACXEH000000000.1
Env16	AAF	Mouse feces	5,371,194	168	5,313,215	50.29	166	210,214	5,003	SRR12593939	JACXEI000000000.1
Env17	AAF	Pig feces	5,652,952	184	5,374,139	50.27	205	148,707	5,089	SRR12593938	JACXEJ000000000.1
Env18	AAF	Pig feces	4,981,227	162	5,401,842	50.29	172	151,080	5,123	SRR12593937	JACXEK000000000.1
Env19	AAF	Pig feces	5,571,233	191	5,370,851	50.27	205	157,855	5,082	SRR12593936	JACXEL000000000.1
Env20	AAF	Pig feces	5,947,112	188	5,494,726	50.31	175	159,923	5,232	SRR12593935	JACXEM000000000.1
Env21	AAF	Manure	5,622,838	188	5,338,140	50.29	177	149,902	5,055	SRR12593934	JACXEN000000000.1
Env22	AAF	Water	5,445,137	179	5,320,497	50.29	177	157,969	5,025	SRR12593933	JACXEO000000000.1
Env23	AAF	Pig feces	7,441,509	249	5,339,774	50.29	177	157,969	5,056	SRR12593911	JACXEP000000000.1
Env24	AAF	Pig feces	5,235,660	167	5,336,902	50.28	176	149,902	5,053	SRR12593910	JACXEQ000000000.1
Env25	AAF	Pig feces	5,809,134	188	5,499,880	50.31	183	157,854	5,231	SRR12593909	JACXER000000000.1
Pig01	23	Pig cecal content	5,841,951	183	5,340,637	50.29	178	157,969	5,050	SRR12593908	JACXES000000000.1
Pig02	23	Pig cecal content	5,866,264	190	5,340,115	50.29	176	149,902	5,057	SRR12593907	JACXET000000000.1
Pig03	23	Pig cecal content	6,267,063	197	5,364,959	50.31	163	157,855	5,088	SRR12593906	JACXEU000000000.1
Pig04	23	Pig cecal content	6,040,007	196	5,408,368	50.35	179	159,727	5,147	SRR12593905	JACXEV000000000.1
Pig05	23	Pig cecal content	6,486,008	216	5,372,102	50.27	192	157,855	5,090	SRR12593904	JACXEW000000000.1
Pig06	23	Pig cecal content	6,268,510	209	5,338,920	50.29	175	149,902	5,056	SRR12593903	JACXEX000000000.1
Pig07	23	Pig cecal content	5,417,785	178	5,337,200	50.28	181	157,969	5,050	SRR12593902	JACXEY000000000.1
Pig08	AAF	Pig feces	6,371,068	213	5,312,973	50.3	156	210,214	5,010	SRR12593900	JACXEZ000000000.1
Pig09	AAF	Pig feces	5,826,200	188	5,320,140	50.29	169	210,112	5,013	SRR12593899	JACXFA000000000.1
Pig10	AAF	Pig feces	6,031,913	184	5,315,000	50.29	163	210,214	5,012	SRR12593898	JACXFB000000000.1
Pig11	AAF	Pig feces	6,741,304	225	5,313,037	50.3	171	203,113	5,000	SRR12593897	JACXFC000000000.1
Pig12	AAF	Pig feces	5,684,078	190	5,313,352	50.29	160	210,214	5,011	SRR12593896	JACXFD000000000.1
Pig13	AAF	Pig feces	5,290,390	181	5,313,653	50.3	165	210,112	5,009	SRR12593895	JACXFE000000000.1
Pig14	AAF	Pig feces	6,331,911	201	5,311,737	50.3	157	210,214	5,008	SRR12593894	JACXFF000000000.1
Pig15	AAF	Pig feces	6,373,650	208	5,292,990	50.3	152	210,802	4,986	SRR12593893	JACXFG000000000.1
Pig16	AAF	Pig feces	6,337,386	205	5,294,068	50.3	161	210,807	4,982	SRR12593932	JACXFH000000000.1
Pig17	AAF	Pig feces	5,823,932	189	5,294,090	50.3	164	210,802	4,977	SRR12593931	JACXFI000000000.1
Pig18	AAF	Pig feces	5,800,520	188	5,331,449	50.32	167	202,338	5,031	SRR12593929	JACXFJ000000000.1
Pig19	AAF	Pig feces	4,583,682	158	5,312,258	50.29	171	201,648	5,000	SRR12593928	JACXFK000000000.1
Pig20	AAF	Pig feces	5,942,592	203	5,425,959	50.2	179	15,7973	5,148	SRR12593927	JACXFL000000000.1
Pig21	AAF	Pig feces	6,566,574	213	5,313,470	50.3	163	21,0112	5,007	SRR12593926	JACXFM000000000.1
Pig22	AAF	Pig feces	5,826,019	189	5,312,857	50.3	170	21,0214	5,011	SRR12593925	JACXFN000000000.1
Pig23	AAF	Pig feces	5,872,913	194	5,315,892	50.3	167	21,0214	5,013	SRR12593924	JACXFO000000000.1
Pig24	AAF	Pig feces	5,221,893	170	5,313,371	50.29	165	21,0802	5,012	SRR12593923	JACXFP000000000.1
Pig25	AAF	Pig feces	6,300,050	221	5,295,743	50.3	172	21,0214	4,982	SRR12593922	JACXFQ000000000.1
Pig26	AAF	Pig feces	8,720,725	327	5,312,878	50.3	163	21,0214	5,005	SRR12593921	JACXFR000000000.1
Pig27	AAF	Pig feces	6,511,839	210	5,292,173	50.3	164	21,0802	4,977	SRR12593920	JACXFS000000000.1
Pig28	AAF	Pig feces	7,510,289	230	5,323,478	50.29	168	188,410	5,032	SRR12593918	JACXFT000000000.1
Pig29	AAF	Pig feces	5,528,538	176	5,322,850	50.29	177	157,969	5,029	SRR12593917	JACXFU000000000.1
Pig30	AAF	Pig feces	6,366,193	202	5,320,117	50.29	176	188,184	5,028	SRR12593916	JACXFV000000000.1
Pig31	AAF	Pig feces	6,557,902	212	5,323,152	50.29	173	157,969	5,028	SRR12593915	JACXFW000000000.1
Pig32	AAF	Pig feces	5,359,720	176	5,323,180	50.29	181	157,969	5,027	SRR12593914	JACXFX000000000.1
Pig33	AAF	Pig feces	6,341,265	204	5,363,886	50.29	166	210,214	5,071	SRR12593913	JACXFY000000000.1
Pig34	AAF	Pig feces	6,096,714	202	5,310,835	50.29	161	210,214	5,007	SRR12593892	JACXFZ000000000.1
Pig35	AAF	Pig feces	6,437,450	210	5,312,008	50.29	164	210,214	5,012	SRR12593891	JACXGA000000000.1
Pig36	AAF	Pig feces	6,751,171	212	5,364,931	50.28	169	210,214	5,073	SRR12593890	JACXGB000000000.1
Pig37	AAF	Pig feces	6,151,804	205	5,352,151	50.27	195	188,184	5,062	SRR12593889	JACXGC000000000.1
Pig38	AAF	Pig feces	5,204,338	174	5,348,858	50.27	196	159,727	5,058	SRR12593969	JACXGD000000000.1
Pig39	AAF	Pig feces	5,448,625	176	5,353,797	50.27	195	157,855	5,067	SRR12593968	JACXGE000000000.1
Pig40	AAF	Pig feces	5,697,729	179	5,335,583	50.28	173	157,969	5,046	SRR12593967	JACXGF000000000.1
Pig41	AAF	Pig feces	6,539,261	211	5,429,978	50.23	170	157,969	5,174	SRR12593966	JACXGG000000000.1

a AAF, the culture collection of Alberta Agriculture and Forestry.

Each isolate was grown in half-strength brain heart infusion broth (Oxoid, Mississauga, ON, Canada) at 35°C for 18 h. The DNA of cultures was extracted using a MasterPure complete DNA and RNA purification kit (Lucigen, Middleton, WI, USA) following the manufacturer’s instructions and was sequenced by Genome Quebec (Montreal, QC, Canada). Sequencing libraries were constructed using a NEBNext Ultra II DNA library prep kit and sequenced for 150 × 2 cycles using an Illumina HiSeq 4000 instrument with an aimed average read depth of >300×. For data analysis, default parameters were used for all software unless otherwise specified. The quality control of raw sequencing reads was performed using FastQC v0.11.8 (30). Trimmomatic v0.39 (31) was used to remove adapter sequences and the sequences with an average quality score of <20 or length of <100 bases. Each genome was assembled using SPAdes v3.14.0 (32) with the size of kmers set at 21, 33, 55, 77, 99, and 127. The quality of the assembled genome was assessed using QUAST v5.0.2 (33). The contigs with a length of <500 bp or coverage of <10× were discarded using a Python script (34). The species identity and serotype for each isolate were confirmed using ECTyper v1.0.0 (https://github.com/phac-nml/ecoli_serotyping). Mauve v2015-02-26 (35) was used to order the remaining contigs by referencing the completed genome of E. coli O157:H7 EDL 933 (36). The genomes were annotated by the National Center for Biotechnology Information using the Prokaryotic Genome Annotation Pipeline v4.13 (37).

### Data availability.

The raw sequencing data and genome sequences have been submitted to GenBank under BioProject number PRJNA661559. The accession numbers are listed in Table 1.
